# The conjugation-associated linear-BAC iterative assembling (CALBIA) method for cloning 2.1-Mb human chromosomal DNAs in bacteria

**DOI:** 10.1038/s41422-024-01063-7

**Published:** 2025-01-06

**Authors:** Li Zhong, Qi Zhang, Ning Lu, Tao Wang, Xiaoli Xue, Zhongjun Qin

**Affiliations:** 1 Key Laboratory of Synthetic Biology, CAS Center for Excellence in Molecular Plant Sciences, Chinese Academy of Sciences, Shanghai, China; 2 State Key Laboratory of Microbial Metabolism, and School of Life Sciences & Biotechnology, Shanghai Jiao Tong University, Shanghai, China

**Keywords:** Genomic analysis, Chromosomes

Dear Editor,

Despite the advancements in the human genome project,^1^ comprehending and manipulating the human genome for practical purposes presents numerous challenges, with the rapid assembly and construction of functional gene clusters spanning millions of base-pairs (Mb) standing out as a pivotal technical obstacle.

DNA assembly stands as a crucial technique for constructing large DNA molecules, up to several hundred kilobases (kb) in size, in vitro.^2,3^ However, larger DNA fragments at the Mb-level are often assembled in vivo due to their susceptibility to fragmentation by shear forces. Currently, the predominant methods for large DNA in vivo assembly rely heavily on the homologous recombination systems in the yeast *Saccharomyces cerevisiae*.^4–6^ Recently, He et al. developed a yeast life cycle-assembly method, successfully assembling a 1.26-Mb human immunoglobulin gene cluster within yeast cells.^7^

*Escherichia coli* is an ideal host for large DNA assembly and chromosome engineering because its low natural homologous recombination activity enhances the genetic stability of human DNA fragments up to 300 kb.^8^ Thus, advancing assembly techniques using *E. coli* is crucial for rapid assembly of complex genomes from humans and other higher organisms. Zurcher et al. developed an assembly method using a circular vector in *E. coli* cells for iterative assembly of a 1.1-Mb human DNA.^9^ However, sequencing results showed that as the DNA size approached the Mb level, the correctly and fully assembled clones dropped to below 10%.

In this study, we have developed a method suitable for assembling Mb-sized human DNA, termed Conjugation-Associated Linear-Bacterial Artificial Chromosome (BAC) Iterative Assembling (CALBIA). By constructing a linear BAC as a carrier and employing a single crossover of homologous recombination, large DNA fragments can be assembled with high efficiency. Utilizing this method, we successfully assembled three Mb-sized human chromosome DNA fragments. Additionally, we generated and utilized linear *E. coli* chromosomes as assembly vectors, assembling a 2.1-Mb human chromosome 18 short arm DNA sequence (18p11.2), which exhibited stable inheritance.

Here, we employed the prokaryotic telomere system TelN-*tos*^10^ to develop linear BAC plasmids as vectors for large DNA assembly (Supplementary information, Fig. S1a). Using the linear BAC vectors, through a single recombination event, the single-stranded DNA form of the linear BAC plasmid B recombined with the homologous segment of the recipient BAC plasmid A, completing the assembly process of B + A. Plasmid incompatibility ensures the formation of a single stable plasmid from the recombination of two BAC plasmids (Fig. 1a).

**Fig. 1:**
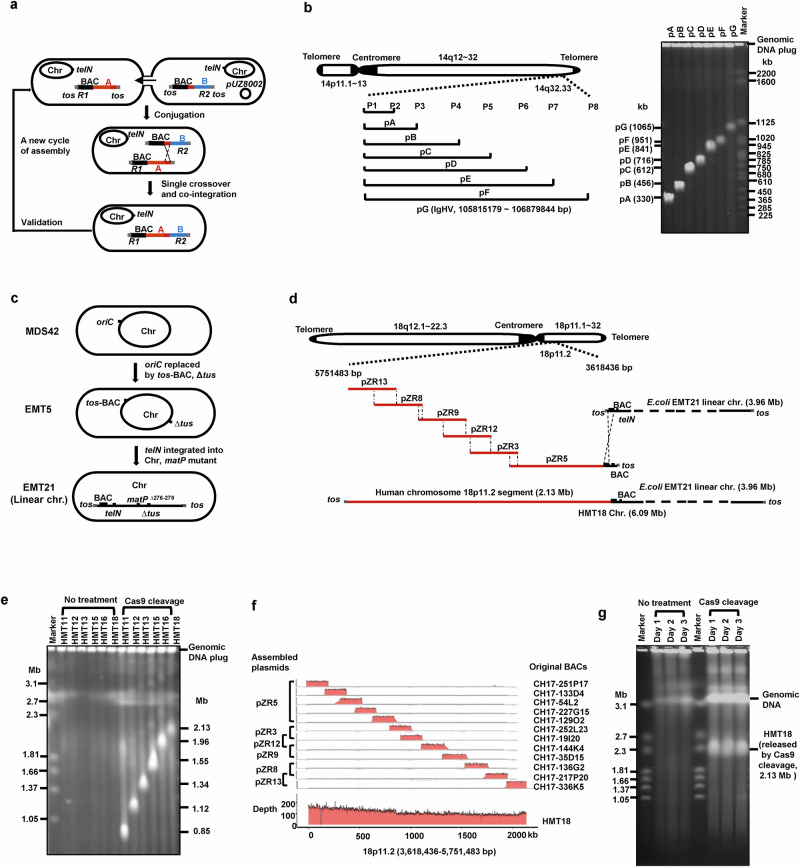
The CALBIA method for the assembly of Mb-scale human genome DNA. **a** Design principle of the conjugation-associated linear-BAC iterative assembly of large DNAs. In each assembly, the strain harboring the helper plasmid pUZ8002 served as the donor, while the other strain acted as the recipient. The linear BAC plasmid in the donor was transferred into the recipient through conjugation, where it recombined with the other linear BAC plasmid at overlapping regions, resulting in the construction of the assembled plasmid. Selective markers R1 and R2 were used to select recombinants. A and B represent human sequences cloned on the BAC plasmid (red and blue bars, respectively). The small red bar to the left of the blue bar B indicates the overlapping region with A. The *tos* site (gray square) and *telN* gene (black dot) are also marked. **b** Assembly of the human *IgHV* gene cluster. The left panel presents a schematic diagram of the BAC plasmid assembly containing the 1.07-Mb *IgHV* gene cluster. The right panel shows PFGE validation of the assembled plasmids pA (330 kb), pB (456 kb), pC (612 kb), pD (716 kb), pE (841 kb), pF (951 kb), and pG (1065 kb). *S. cerevisiae* sixteen chromosomes were used as markers (Bio-Rad, 1703605). **c** Schematic diagram of the construction of the *E. coli* strain EMT21. To construct the strain EMT5, the *oriC* of the *E. coli* MDS42 chromosome was replaced with the *tos*-BAC cassette containing the BAC replication origin, partition system, and *tos* site, followed by the deletion of the *tus* gene. When the TelN expression plasmid was introduced into EMT5 (*tus* knockout) by transformation, it took a week for a dozen very small colonies to appear on the plate. After moving the TelN expression cassette from the plasmid to its chromosome, EMT21 was constructed, and its chromosome was validated as linear. Through genome sequencing, a 4-bp deletion at positions 276–279 in *matP* was identified, resulting in a frameshift mutation. **d** Schematic diagram of the assembly of 2.13-Mb human DNAs on the linear chromosome of *E. coli*. Assembled plasmids pZR5 (845 kb), pZR3 (311 kb), pZR12 (423 kb), pZR9 (438 kb), pZR8 (424 kb), and pZR13 (398 kb) were iteratively assembled and integrated into the linear chromosome of EMT21, resulting in strain HMT18. **e** Verification of assembled human DNA released by Cas9 cleavage using PFGE. The linear chromosomes with integrated DNA assemblies in strains HMT11, HMT12, HMT13, HMT15, HMT16, and HMT18 are very large, ranging from 5–6 Mb, and mostly remain in the gel block under the electrophoresis conditions. Cas9 cleavage released the assembled DNA, which ranged from 0.85 to 2.13 Mb and could be separated by PFGE. The chromosome of *Hansenula wingei* was used as a marker (Bio-Rad, 1703667). **f** Validation of the integrity of the assembled 2.13-Mb human DNA through high-throughput sequencing. The lower part shows the sequencing coverage of the insert in HMT18, while the upper part displays the coverage of the 12 original BACs. Read coverage (*y*-axis) is plotted against the position in base pairs from the start of the 2.13-Mb assembly. **g** PFGE validation of the genetic stability of the integrated 2.13-Mb assembled DNA. The integrated DNA was released by Cas9 nuclease from HMT18. The size and integrity of the assembly remained consistent after serial cultivation for three days, as shown in the PFGE gel.

The experimental workflow of the CALBIA method is rapid, requiring only 1–2 h of conjugation transfer and overnight culture of transconjugants for screening, allowing assembly completion within 1 day (Supplementary information, Fig. S1b). Furthermore, linear vectors facilitate easy separation and verification of the assembled linear DNA fragments by pulsed-field gel electrophoresis (PFGE) based on DNA size, a process that takes only 1–2 days.

To demonstrate the robust capability of the CALBIA method in assembling large segments of human DNA, we selected the immunoglobulin heavy chain variable region (IgHV) gene cluster for assembly. Spanning at the megabase level and comprising nearly a hundred repetitive units, this cluster holds significant importance in specificity and diversity of human antibodies.^11^ We utilized the CALBIA method to consecutively assemble seven BAC plasmids, each containing 150–200 kb of the IgHV gene cluster, and successfully assembled the complete gene cluster of IgHV in pG, with a total size of 1.07 Mb (Fig. 1b).

With this method, we assembled two consecutive ultra-large DNAs, pZR6 and pZR11, containing Mb-scale fragments from the 18p11.2 region in the human genome (Supplementary information, Fig. S2). PFGE validation indicated that up to 60% of colonies contained the positive DNA band size (Supplementary information, Table S1).

It is noteworthy that as the assembled DNA approached 1 Mb in size, the detectable DNA bands in PFGE gradually diminished (Fig. 1b; Supplementary information, Fig. S2b). To tackle the challenge of genetic instability, we constructed a linearized *E. coli* chromosome to serve as an assembly vector. The engineered *E. coli* strain EMT21 features a prokaryotic telomere system TelN-*tos* and a BAC replication and distribution system near one end of the linear chromosome (Fig. 1c). Verification of genome digestion and validation through genome sequencing of the EMT21 strain are provided in Supplementary information, Fig. S3 and Supplementary information, Table S4, respectively.

Utilizing the 5.5 kb BAC replication and distribution elements present on the EMT21 chromosome as arms for homologous recombination, free BAC plasmids containing Mb-scale human DNA can be seamlessly integrated into the linear chromosome of *E. coli* in a single step via conjugation transfer (Supplementary information, Fig. S4a). Integration into the chromosome significantly increases the genetic stability of the heterologous Mb-scale ultra-large DNAs, pG and pZR11, which harbor 1.07 Mb and 1.18 Mb of human DNA, respectively, within *E. coli* (Supplementary information, Fig. S4b, c). In comparison, a gradual loss of assembled extrachromosomal ultra-large DNAs, pG or pZR11, was observed within the bacterial population, even when cultivated under antibiotic selection. Some smaller plasmid DNA bands appeared at the bottom of the gel map after 3 days of passage cultivation, while the DNA bands corresponding to the original plasmid size became fainter.

We further assembled larger DNA segments from human chromosome 18 using the linear EMT21 chromosome as a vector. We initiated this process by selecting the BAC plasmid pZR5, which had already assembled 845 kb of human DNA, as its genetic stability surpassed that of pZR6 containing 1.12 Mb of human DNA. In a single step via conjugation transfer, pZR5 was integrated into the linear chromosome of EMT21. Following this, through five rounds of consecutive CALBIA assembly, we successfully synthesized > 2 Mb of human DNA in *E. coli*. The resultant *E. coli* strain, HMT18, boasted a chromosome size of 6.09 Mb, housing 2.13 Mb of human DNA and 3.96 Mb of *E. coli* DNA (Fig. 1d, e). Multiple PCR and PFGE validations revealed that the accuracy of the assemblies ranged from 67% to 100% (Supplementary information, Table S2), with no decrease in efficiency observed as the size of the assembled DNA increased.

Whole genome sequencing further verified the integrity of the four Mb-scale DNAs (pG: 1.1 Mb; pZR6: 1.1 Mb; pZR11: 1.2 Mb; HMT18: 2.1 Mb). For pG, whole-genome sequencing revealed two large deletions in six PFGE-positive colonies: a 37.7-kb deletion (260–297 kb) and a > 60-kb deletion (~879–949 kb). Similar deletions were also presented in the assembly plasmid pF and the starting BAC plasmids (659B19 and 2366K3). We attempted to correct the second deletion by assembling of pF and p8 (which overlaps by > 100 kb with pF and lacks the second deletion), but only one in the six sequenced colonies showed recovery of the second deletion, with no SNP or insertion-deletion (InDel) in comparison to pF and p8. Therefore, the final assembly efficiency for pG was calculated as [(6/10) × (1/6)] = 10%. The sequencing data are provided in Supplementary information, Fig. S5 and Supplementary information, Table S5.

For pZR6 and pZR11, 9 and 7 PFGE-positive colonies, respectively, were sent for sequencing. No large deletions, duplications, SNPs, or InDels were found, yielding final assembly efficiencies of 90% [(9/10) × (9/9)] for pZR6 and 70% [(7/10) × (7/7)] for pZR11. The sequencing data are provided in Supplementary information, Figs. S6 and S7.

For HMT18, ten colonies that were positive in both PCR and PFGE validation were sequenced. Two large deletions were found in HMT18-3 (9.3 and 55.6 kb), and a large duplication was detected in HMT18-7. In the remaining eight colonies, the five 1-base deletions and one 4-base insertion were found in AT-rich repeat sequences. These variations, caused by polymerase slippage, are common in AT-rich regions and were not considered assembly errors, as previously reported by Zurcher et al. ^9^ The final assembly efficiency for HMT18 was calculated as [(10/13) × (10/10) × (8/10)] = 61.5%. The sequencing data and variations are detailed in Supplementary information, Fig. S8 and Supplementary information, Table S6. Four HMT18 colonies were further selected for de novo sequencing. In the 2.1-Mb sequence, we identified four 1-base deletions and one 3-base deletion within the AT-rich repeat region (Supplementary  information, Table S7).

The 2.13-Mb human genome DNA assembled on a linear chromosome demonstrates preferable genetic stability, with clear target bands visible in PFGE after 3 days of passage (Fig. 1g). The *E. coli* strain HMT18 has a generation time of 52.1 min, equating to 83 generations over 3 days (Supplementary information, Fig. S9). Sequence changes in two HMT18 clones were evaluated using de novo sequencing across 3 days of passaging. One colony, HMT18-14, showed no sequence changes, while the other colony, HMT18-13, exhibited 10 instances of single- or double-base deletions or insertions in the AT-rich region. Detailed variations are provided in Supplementary information, Table S8.

In comparison to previously reported methods for assembling Mb-scale human DNA (Supplementary information, Table S3), the CALBIA method developed in this study offers distinct advantages in both assembly size (2.13 Mb) and genetic stability (up to 3 days) of human genome DNA. We anticipate that this method will emerge as a valuable technique for the assembly of complex super-large DNA, thereby facilitating rapid and efficient genome assembly and enabling new functional applications in higher eukaryotes.

It is worth mentioning that the CALBIA method does not necessitate the introduction of the Cas9 system^12^ or the Lambda-Red homologous recombination system.^13^ This eliminates the risk of erroneous recombination involving small repetitive sequences following Cas9 cleavage. Consequently, this method is particularly well-suited for assembling human genome DNA containing repetitive sequences.

Similar to the genetic instability observed in Mb-scale human DNA assemblies using yeast artificial chromosome,^7^ our study found that using BAC plasmids also exhibits instability after assembling Mb-scale DNAs. The linear chromosome provides an ideal platform for large DNA assembly. However, constructing such a linear BAC-chromosome in *E. coli* poses significant challenges. While replacing the native replication origin *oriC* with the BAC origin and *parABS* did not impact cell growth, linearization near the replication origin by the TelN-*tos* system resulted in severely compromised cell growth (Supplementary information, Fig. S3c). This alteration shifts chromosome replication from bidirectional to unidirectional, disrupting the replication balance. During the construction of EMT21, we found that the *tus* gene knockout and *matP* gene mutation were critical. The linearization nearby the origin can only be achieved by deletion of *tus*, which is a key regulator of bidirectional replication forks in *E. coli*.^14^ Additionally, genome sequencing of EMT21 revealed a 4-bp deletion at positions 276–279 in *matP*, resulting in a frameshift mutation. MatP is an essential regulator of *E. coli* chromosome segregation.^15^ Linearization near the origin places the MatP recognition site, *matS*, in the middle of the linear chromosome rather than at the ends. MatP mutations may prevent premature segregation of unidirectional duplicated linear chromosomes.

Utilizing the constructed linear *E. coli* chromosome as a vector largely resolves the issue of genetic stability in assembling human DNA. This represents a pivotal advancement, enabling the assembly and stable inheritance of the longest human super-large DNA fragments (longer than 2 Mb) in bacteria.

## Supplementary information

Supplementary Information

## Data Availability

All sequencing data in this paper have been deposited in NCBI with the accession numbers of PRJNA1098915 (HMT18), PRJNA1098934 (EMT21), PRJNA1097768 (CH17-251P17), PRJNA1097783 (CH17-133D4), PRJNA1097800 (CH17-54L2), PRJNA1097810 (CH17-227G15), PRJNA1097835 (CH17-129O2), PRJNA1098439 (CH17-252L23), PRJNA1098493 (CH17-19I20), PRJNA1098503 (CH17-144K4), PRJNA1098576 (CH17-35D15), PRJNA1098870 (CH17-136G2), PRJNA1098873 (CH17-217P20), PRJNA1098885 (CH17-336K5), PRJNA1159494 (pZR3), PRJNA1159547 (pZR5), PRJNA1158873 (nine pZR6 colonies), PRJNA1159598 (pZR7), PRJNA1160229 (pZR10), PRJNA1161133 (seven pZR11 colonies), PRJNA1159480 (HMT16), PRJNA1159483 (pZR13), PRJNA1158507 (eighteen HMT18 samples), PRJNA1162302 (CTD-2572O2, RP11-659B19, RP11-413L20, RP11-72N10, CTD-3074B5, CTD-2195P5, CTD-2366K3), PRJNA1160705 (p8), PRJNA1160241 (pF), and PRJNA1161033 (six pG colonies).
