# Supplementary material for: The conjugation-associated linear-BAC iterative assembling (CALBIA) method for cloning 2.1-Mb human chromosomal DNAs in bacteria
Source: Cell Res. 2025 Jan 6;35(4):309–12. doi: 10.1038/s41422-024-01063-7 (PMC11958681; doi:10.1038/s41422-024-01063-7)
Supplement: Supplementary file 1 — Supplementary Information [file 41422_2024_1063_MOESM1_ESM.pdf]

Supplementary Information

CALBIA (Conjugation-Associated Linear BAC Iterative Assembling) method for cloning 2.1-Mb human chromosomal DNA in *Escherichia coli*

Li Zhong[1†], Qi Zhang[1†], Ning Lu[1†], Tao Wang[1], Xiaoli Xue[2*], Zhongjun Qin[1*]

[1] Key Laboratory of Synthetic Biology, CAS Center for Excellence in Molecular Plant Sciences, Chinese Academy of Sciences, Shanghai 200032, P. R. China

[2] State Key Laboratory of Microbial Metabolism, and School of Life Sciences & Biotechnology, Shanghai Jiao Tong University, Shanghai 200240, P. R. China

[†] Joint Authors. These authors contributed equally: Li Zhong, Qi Zhang, Ning Lu.

[*] To whom correspondence should be addressed. Email: qin@cemps.ac.cn.

Correspondence may also be addressed to xlxue@sjtu.edu.cn.

Supplementary Figures, Tables, Materials and Methods.

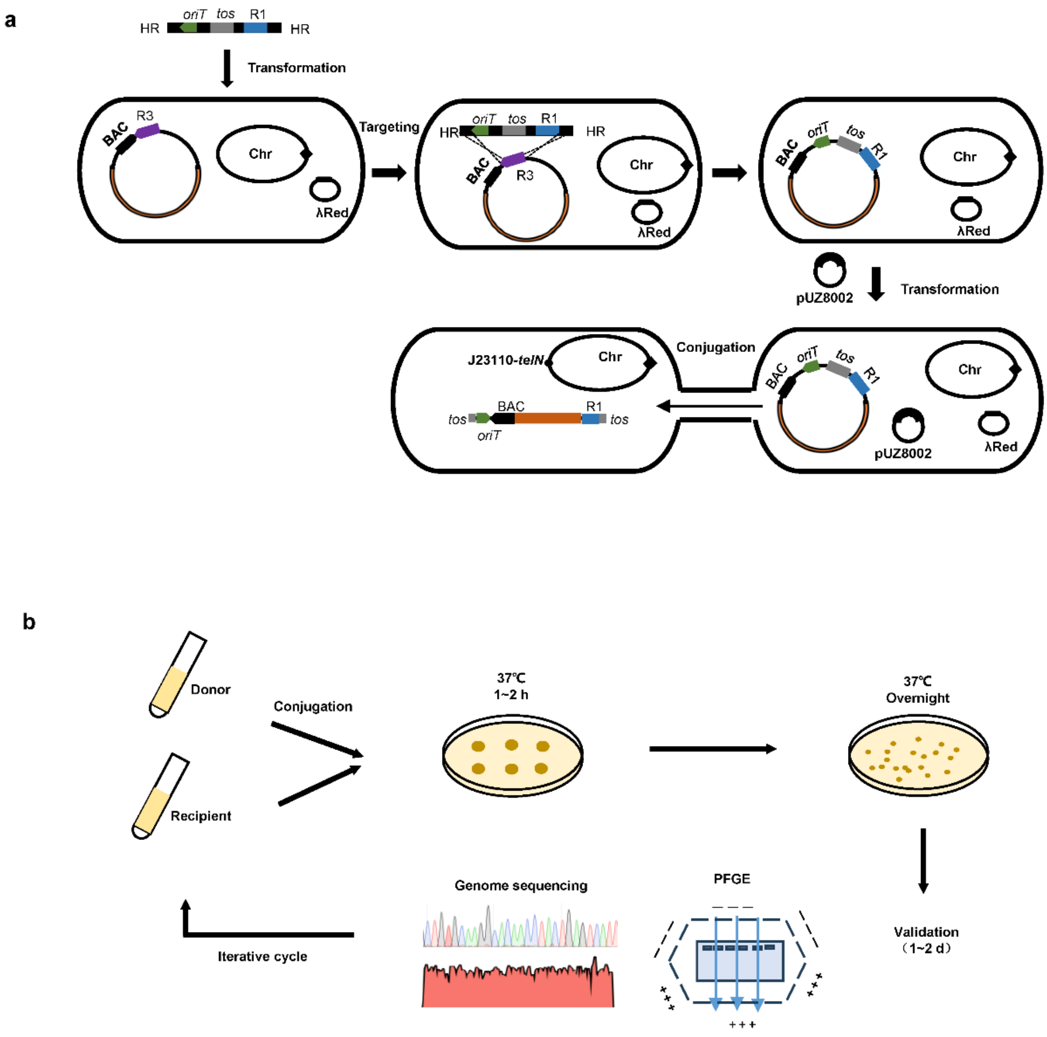

Figure S1. Detailed depiction of the CALBIA procedure. (a) Schematic diagram of BAC modification. The chloramphenicol resistance marker R3 on the BAC vector was chosen as the targeting site, and the components containing *oriT-tos* and the ampicillin resistance marker R1 were introduced into *E. coli* MDS42 (which contains the λ-Red recombination system). The R3 region was replaced via homologous recombination by using the λ-Red system, and the positive colonies were selected by screening for R1 resistance. The T4SS helper plasmid pUZ8002 was then introduced to facilitate the transferring of the circular plasmid to the recipient strain MDS42 (*telN*), completing the linearization of the plasmid. **(b)** Experimental workflow of the iterative CALBIA cycle.

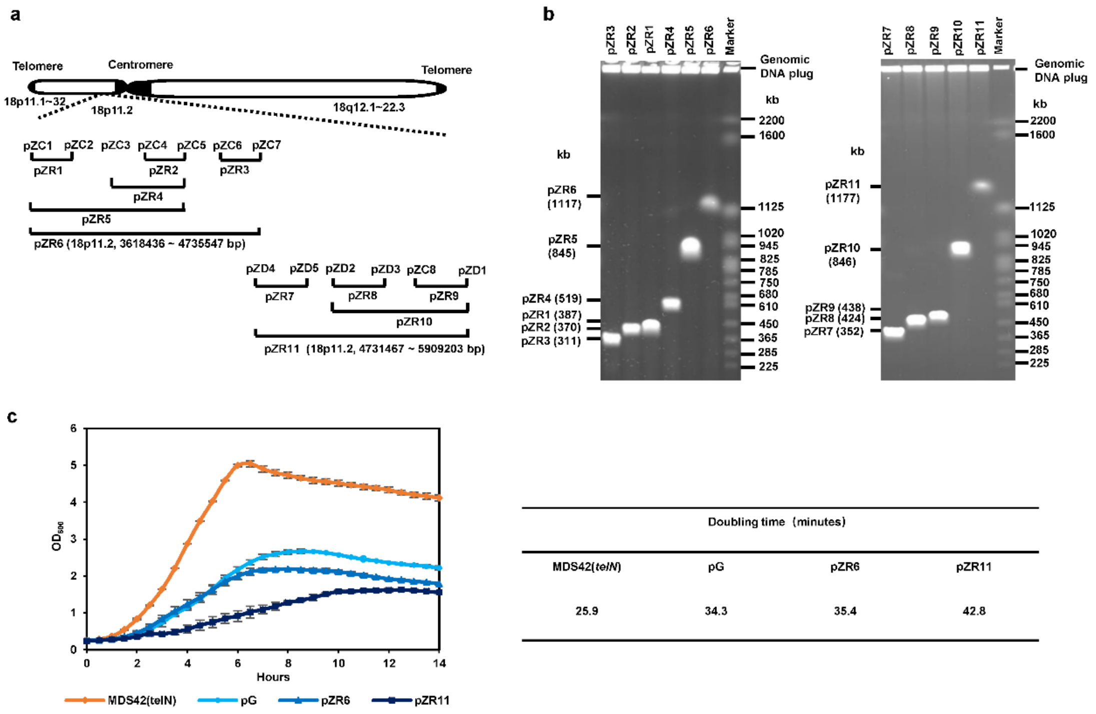

Doubling time (minutes)			
MDS42(*telN*)	pG	pZR6	pZR11
25.9	34.3	35.4	42.8

Figure S2. CALBIA method for the assembly of Mb-scale human genome DNA. (a) Schematic diagram depicting the parallel assembly of two Mb-scale DNA fragments (1.12, 1.18 Mb) within the 18p11.2 region of human chromosome 18. **(b)** PFGE validation of the DNA size during the assembly process of pZR6 and pZR11. **(c)** Growth curves of strains carrying pG, pZR6 and pZR11. Overnight cultures were diluted with fresh LB medium to an OD_{600} of 0.1 and cultivated in the high-throughput microbial growth curve analysis system MicroScreen HT. The absorbance at OD600 was measured every 20 minutes. Six clones from each strain were taken for growth curve experiments.

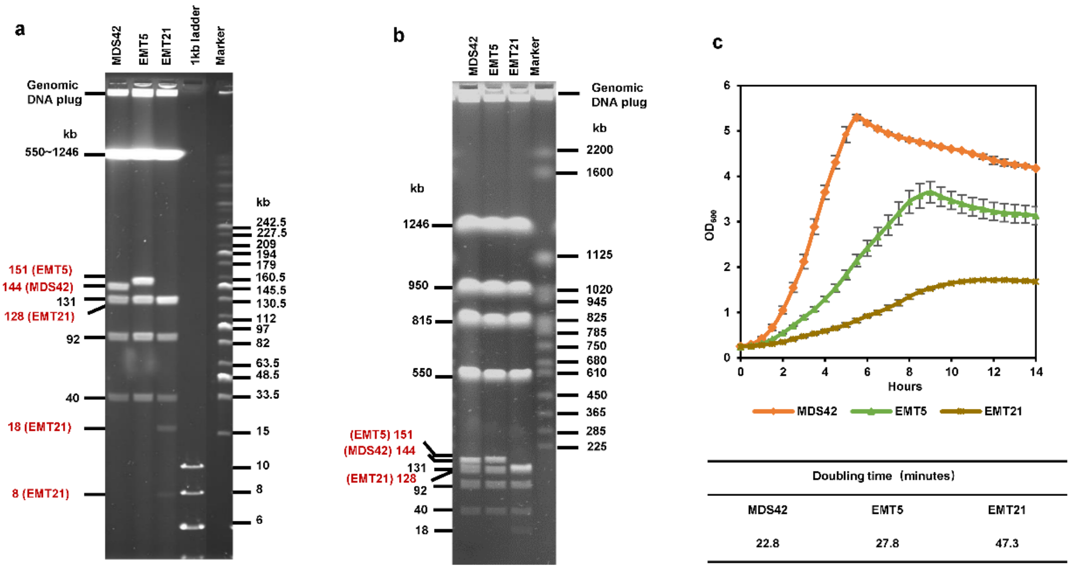

Doubling time (minutes)		
MDS42	EMT5	EMT21
22.8	27.8	47.3

Figure S3. Validation of circular chromosome of strain EMT5 and linear chromosome of EMT21. Restriction endonuclease *Avr*II was used to digest chromosome DNA of wildtype strains MDS42, EMT5 and EMT21. Comparing to 144 kb DNA band of MDS42, EMT5 produced a 151 kb DNA band and EMT21 produced three DNA bands, including 128, 18 and 8 kb in sizes. In EMT5, a 151 kb DNA band was the result of replacing *oriC* (554 bp) with *apra-tos-ori*$_{BAC}$ cassette (7561bp). In EMT21, *Avr*II site in *telN* nucleotide sequence and TelN nuclease cutting at *tos* locus led to produce 128, 18 and 8 kb DNA bands. In order to manifest differences clearly, two PFGE procedures were applied to separate different size DNA bands. (**a**) The MDS42, EMT5 and EMT21 chromosomal DNA digested with *Avr*II followed by PFGE analysis. PFGE procedure for separating smaller DNA bands was initial switch time 1 second, final switch time 25 seconds, run for 18 hours, at angle 120 degrees and voltage 6V/cm. **(b)** The MDS42, EMT5 and EMT21 chromosomal DNA digested with *Avr*II followed by PFGE analysis. Procedure for separating larger DNA bands was initial switch time 60 second, final

switch time 120 seconds, run for 24 hours, at angle 120 degrees and voltage 6V/cm. **(c)** Growth curves of the MDS42, EMT5 and EMT21 strains. Six clones from each strain were taken for growth curve experiments.

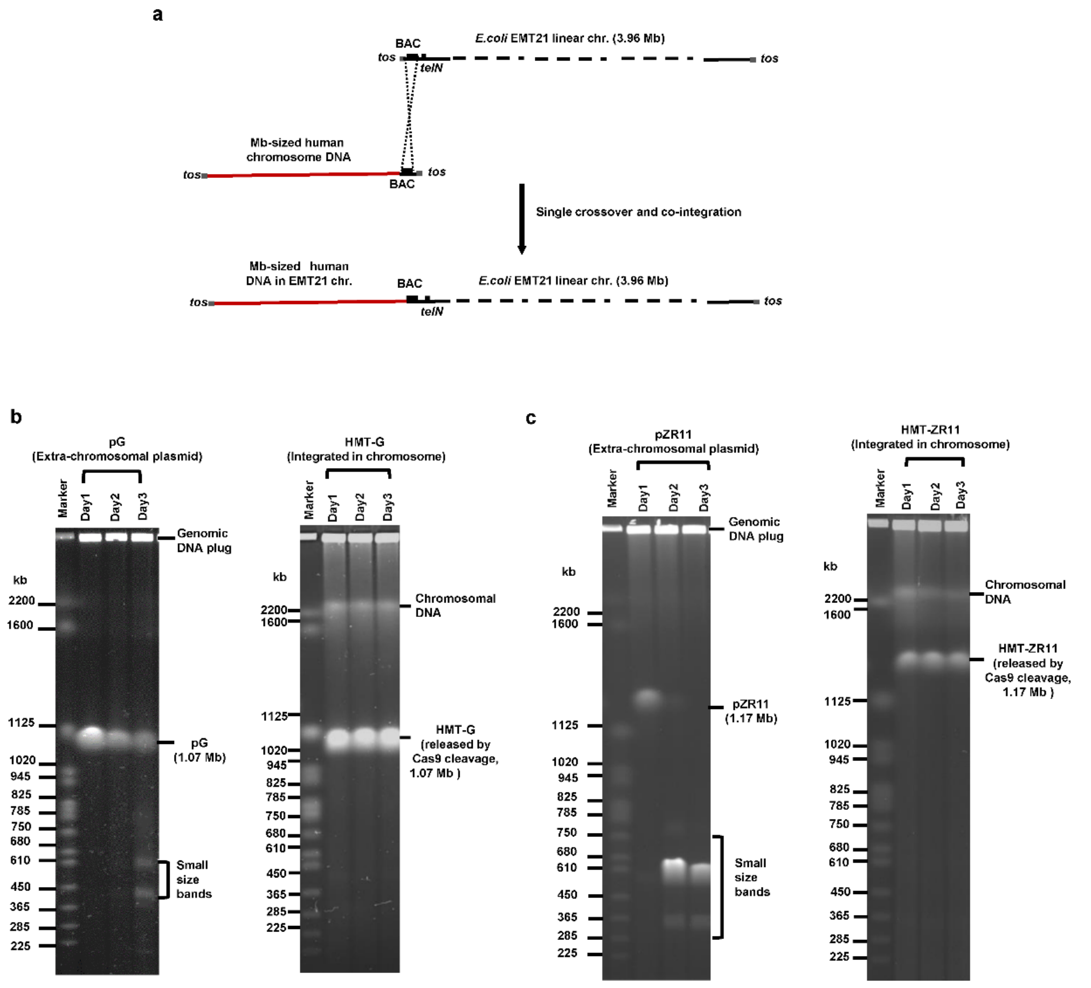

Figure S4. Comparison of the genetic stability of Mb-scale human DNA assembled in free and integrated states. (a) Schematic diagram depicting one-step integration of Mb-scale human DNA. **(b, c)** PFGE validation of the genetic stability of free and integrated Mb-scale assembled DNA, pG (b), and pZR11 (c).

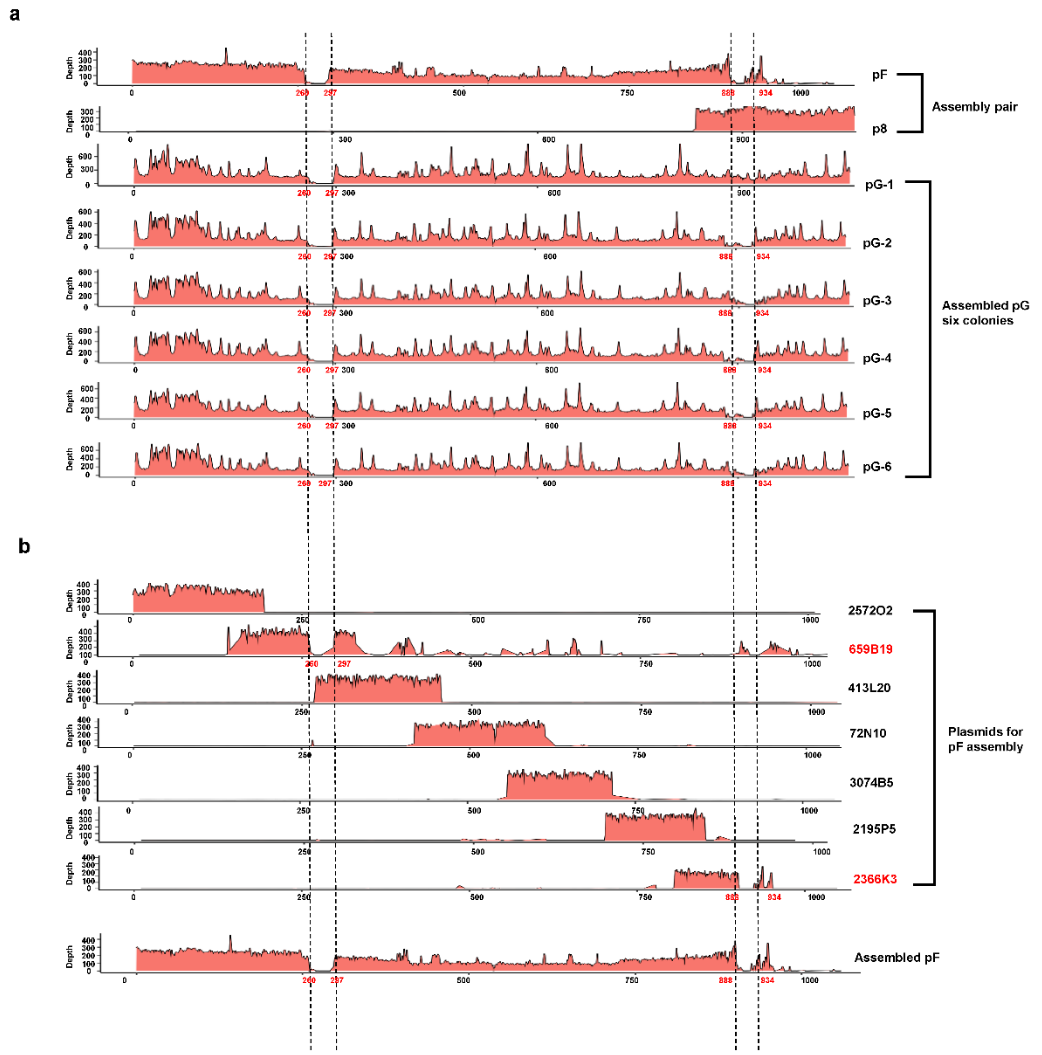

Figure S5. Validation of the integrity of pG (a) and its source plasmids (b) for assembly through whole genome sequencing.

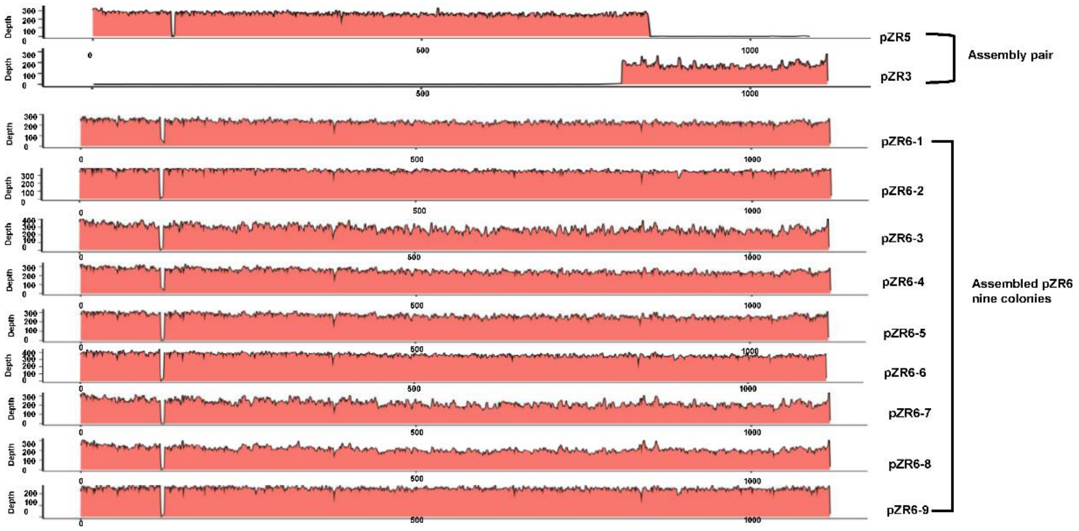

Figure S6. Validation of the integrity of pZR6 and its source plasmids for assembly through whole genome sequencing.

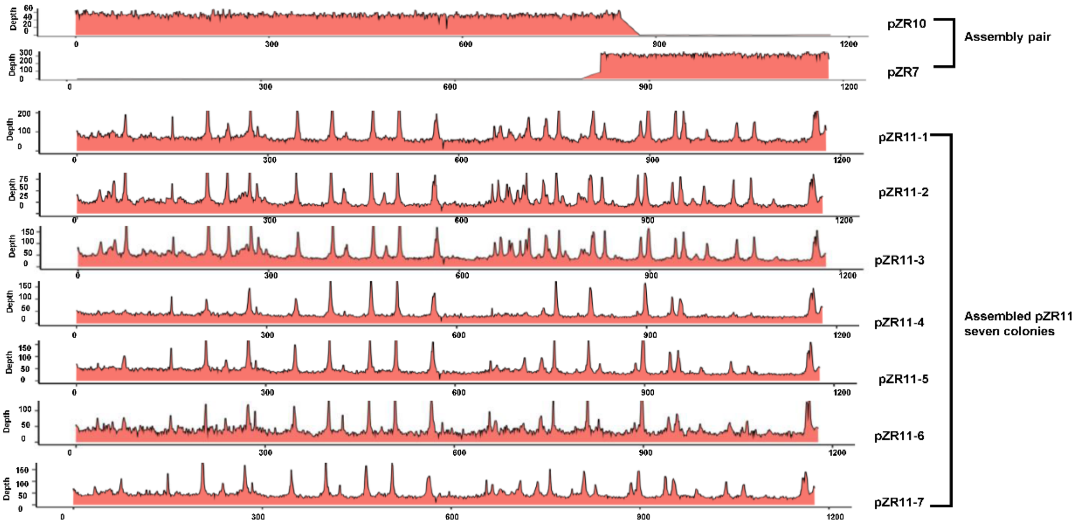

Figure S7. Validation of the integrity of pZR11 and its source plasmids for assembly through whole genome sequencing.

a

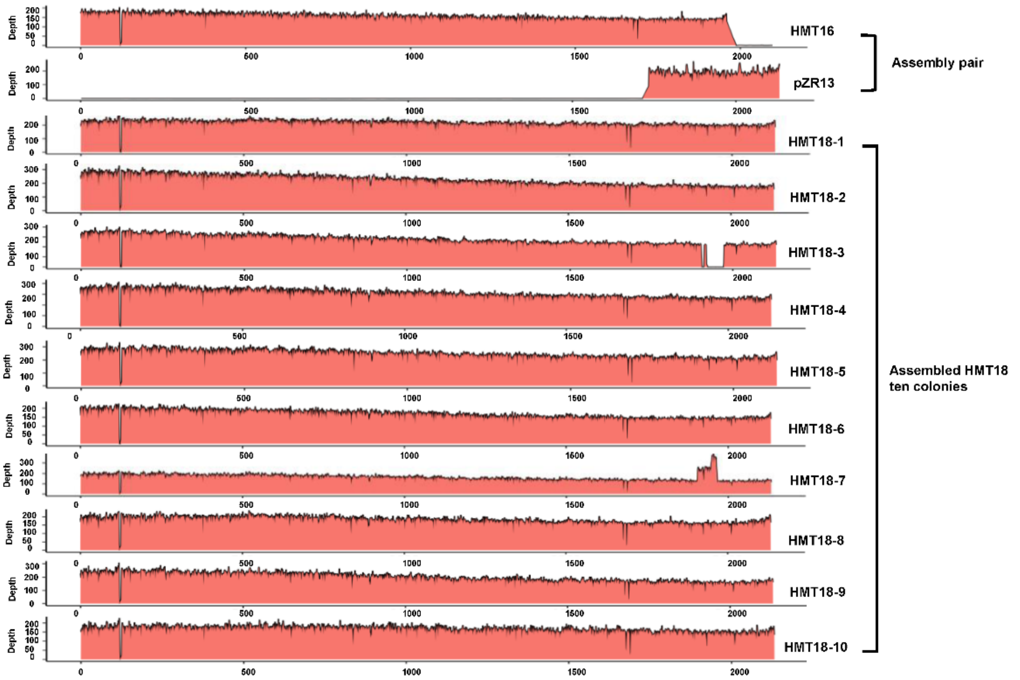

b

Large deletions or duplications in HMT18 compared to the assembly pair

	Deletion		Duplication	
HMT18 colony	Position (bp)	Length (bp)	Position (bp)	Length (bp)
HMT18-3	1903701-1913000,<br>1918201-1973800	9300,<br>55600	/	/
HMT18-7	/	/	1925801-1965500	39700

Figure S8. Validation of the integrity of HMT18 through whole genome sequencing. (a) Whole genome mapping of the 10 colonies of the 2.13 Mb-sized human DNA sequence within HMT18 comparing with its assembly pair HMT16 and pZR13. **(b)** Large deletions or duplications in different colonies containing the assembled HMT18.

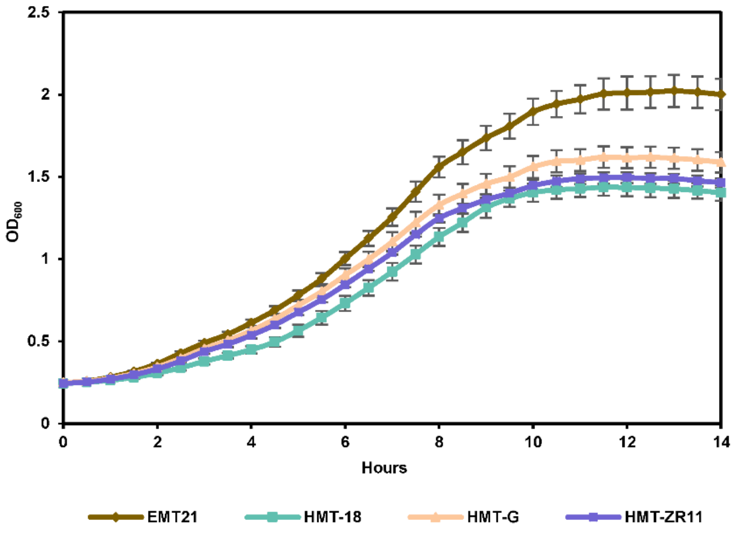

Doubling time（minutes）			
EMT21	HMT-G	HMT-ZR11	HMT18
46.1	49.5	51.7	52.1

Figure S9. Growth curves of strains with pG, pZR11 and 2.13Mb human chromosome 18 sequence integrated into the linear chromosome. Six clones from each strain were taken for growth curve experiments.

Table S1. Assembly information and efficiency of Mb-scale human DNA using the linear BAC plasmid as vectors.

Assembled DNA (kb)	Assembly pairs (kb)		Overlapping (kb)	Number of conjugants [b]	PFGE positive rates[a]
pG (assembled 14q32.33 from 105,815,179 to 106,879,844 bp)					
pA (330)	p1 (193)	p2 (170)	33	12	11/11
pB (456)	pA (330)	p3 (185)	59	230	8/8
pC (612)	pB (456)	p4 (193)	37	212	7/7
pD (716)	pC (612)	p5 (157)	53	130	5/6
pE (841)	pD (716)	p6 (146)	21	91	8/8
pF (951)	pE (841)	p7 (145)	35	118	3/5
pG (1065)	pF (951)	p8 (232)	118	450	3/5
pZR6 (assembled 18p11.2 from 3,618,436 to 4,735,547 bp)					
pZR1 (387)	pZC1 (212)	pZC2 (207)	32	$1x10^4$	5/5
pZR2 (370)	pZC4 (197)	pZC5 (205)	32	$1x10^3$	5/5
pZR3 (311)	pZC7 (204)	pZC6 (207)	100	$8x10^5$	10/10
pZR4 (519)	pZR2 (370)	pZC3 (211)	62	$3x10^3$	6/6
pZR5 (845)	pZR4 (519)	pZR1 (387)	61	$1x10^3$	9/10
pZR6 (1117)	pZR5 (845)	pZR3 (311)	39	$1x10^3$	10/10
pZR11 (assembled 18p11.2 from 4,731,467 to 5,909,203 bp)					
pZR7 (352)	pZC8 (194)	pZD1 (201)	43	641	4/4
pZR8 (424)	pZD2 (220)	pZD3 (224)	20	182	4/4
pZR9 (438)	pZD4 (223)	pZD5 (233)	18	199	4/4
pZR10 (846)	pZR9 (438)	pZR8 (424)	16	134	6/8
pZR11 (1177)	pZR10 (846)	pZR7 (352)	21	70	6/8

[a] To verify correct assembly, five or more colonies from each assembly experiment were

selected directly subjected to PFGE analysis without prior PCR validation. The positive rate was calculated based on the PFGE analysis.

[b] For the assembly of pG and pZR11, only ten percent of the recipient bacteria (OD_{600} = 0.2) were used in conjugation experiments, resulting in less conjugants.

Table S2. Assembly information and efficiency of 2.13 Mb human DNA using the linear BAC-chromosome as a vector.

Assembled DNA (kb)	Assembly pairs (kb)		Overlapping (kb)	Number of conjugants	Positive rates validated by PCR and PFGE[a]
HMT18 (assembled 18p11.2 from 3,618,436 to 5,751,483 bp)					
HMT11 (845)	EMT21	pZR5 (845)	6.7	$1x10^3$	5/5
HMT12 (1117)	HMT11 (845)	pZR3 (311)	39	$3x10^4$	2/3
HMT13 (1336)	HMT12 (1117)	pZR12 (423)	204	$4x10^3$	5/5
HMT15 (1551)	HMT13 (1336)	pZR9 (438)	223	$1x10^4$	5/5
HMT16 (1959)	HMT15 (1551)	pZR8 (424)	16	$1x10^4$	19/20
HMT18 (2133)	HMT16 (1959)	pZR13 (398)	224	$1x10^3$	10/13

[a] Ten or more colonies from each assembly experiment were selected and subjected to PCR

verification and PFGE analysis to confirm correct assembly. The assembly positive rate was calculated by multiplying the PCR positive rate and PFGE positive rate.

Table S3. Comparison of methods for Mb-sized human genome assembly.

**Assembly method**	**Assembly host**	**Size of assembled DNAs**	**Assembly accuracy**	**Assembly time per round**	**Genetic stability**	**Reference**
**CALBIA** (Conjugation-Associated Linear BAC Iterative Assembling)	*Escherichia coli*	2.13 Mb,<br>1.12 Mb,<br>1.18 Mb,<br>1.07 Mb	61.5%[a]<br>90%[b]<br>70%[b]<br>10%[b,c]	2~3 days	Stable up to 3 days	This study
**YLC-assembly** (assembly via Yeast Life Cycle)	*Saccharomyces cerevisiae*	1.26 Mb	60~100%	11-13 days	Segmental deletions	He et al.[1]
**BASIS** (Bacterial Artificial chromosome Stepwise Insertion Synthesis)	*Escherichia coli*	1.1 Mb	8.60%	2 days	NA	Zurcher et al.[2]

Assembly accuracy was calculated as follows:

[a] The accuracies of PCR (10/13), PFGE (10/10), and sequencing (8/10) were multiplied, resulting in an overall accuracy of 61.5%.

[b] For pG, pZR6, and pZR11, PFGE validation was performed directly without PCR validation due to the relative ease of assembly. The final assembly efficiency for these DNAs was calculated by multiplying the PFGE positive rate and the sequencing positive rate, yielding

90% for pZR6 [(9/10) × (9/9)], and 70% for pZR11 [(7/10) × (7/7)], and 10% for pG [(6/10) × (1/6)].

[c]The 1.07 Mb human IgHV gene cluster assembled in pG, contains numerous repetitive units, which may have contributed to the increased recombination in this region, leading to a low assembly accuracy.

Table S4. Whole genome sequencing validation of the *E. coli* strain EMT21.

Location in EMT21 genome	Mutation types	Reference sequences	Mutations	Descriptions
1161260	SNP	T	G	Amino acid replacement at 71th of gene *PagP*, Tyr replaced by Asp
40016	SNP	G	T	Amino acid replacement at 231th of CDS of threonine ammonia-lyase, Asp replaced by Tyr
137187	SNP	T	A	Amino acid replacement at 383th of oxygen-independent coproporphyrinogen III oxidase, Leu replaced by Gln
1512469	InDel	AATCG	A	Deletion of 276-279bp of *matP* causing frameshift

SNP: single nucleotide polymorphism

InDel: insertion and deletion

Table S5. Whole genome sequencing validation of pG and the source plasmids for assembly.

Plasmid name	Description	Large deletion region 1 [a]		Large deletion region 2 [a, b]	
		Position (bp)	Size(bp)	Position (bp)	Size(bp)
659B19	Source plasmids for pF assembly	259901-297600	37699	NA	NA
2366K3		NA	NA	901101-	33399

				934500	
p8	Source plasmids	NA	NA	/	/
pF	for pG assembly	259901-297600	37699	888201-934500	46399
pG-1 [c]	Colonies of assembled pG	259901-297600	37699	/	/
pG-2		259901-297600	37699	879048-944993	65945
pG-3		259901-297600	37699	888024-949317	61293
pG-4		259901-297600	37699	877246-944134	66888
pG-5		259901-297600	37699	879073-944074	65001
pG-6		259901-297600	37699	886879-949482	62603

[a] All variants were further subjected to PCR amplification and sequencing.

[b] NA: not applicable. /: no variation.

[c] No SNPs or InDels were found in pG-1 compared to the plasmid pair (pF and p8) for pG assembly.

Table S6. Whole genome sequencing validation of HMT18 and the source plasmids for assembly.

Plasmid name	Description	Variants information [a]					
		1:<br>deletion A<br>TAAAAA AAAAA → TAAAAA AAAA<br>Position: 887064 bp	2[b]:<br>deletion A<br>GAAAAA AAAAA → GAAAAA AAAA<br>Position: 1240663 bp	3:<br>deletion A<br>CAAAAA AAAA → CAAAAA AAA<br>Position: 1291912 bp	4:<br>deletion T<br>CTTTTTT TTTTT → CTTTTTT TTTT<br>Position: 1540637 bp	5:<br>deletion A<br>GAAAAA AAAAAA A→ GAAAAA AAAAAA<br>Position: 1883778 bp	6:<br>insertion TTAT<br>ATTATTTATTTA TTTATTTATTTA TTTATTTATTTA TTTAT→ ATTATTTATTTA TTTATTTATTTA TTTATTTATTTA TTTATTTAT<br>Position: 1942413 bp
HMT16	Plasmid pair for HMT18 assembly	/	/	/	/	/	/
pZR13		NA	NA	NA	NA	NA	NA
HMT18-1	Colonies of assembled HMT18	/	Deletion A	/	/	/	Insertion TTAT
HMT18-2		/	Deletion A	/	/	/	/
HMT18-4		Deletion A	Deletion A	/	/	Deletion A	Insertion TTAT
HMT18-5		/	Deletion A	/	/	/	/
HMT18-6		/	/	/	/	/	/
HMT18-8		/	/	/	Deletion T	/	/
HMT18-9		/	/	Deletion A	/	/	/
HMT18-10		/	/	/	/	/	/

[a] All variants were further subjected to PCR amplification and sequencing.

[b] Variant 2, deletion of one A in GAAAAAAAAAA at position 1240663 was also observed in both the assembled pZR11 and its last-step assembly plasmid pZR10.

Table S7. *De novo* genome sequencing validation of HMT18 by long-read PacBio sequencing and next-generation sequencing.

**Plasmid name**	**Description**	**Variants information [a]**				
		1: deletion T CTTTTTTTTTTTT→ CTTTTTTTTTTT Position: 478029 bp	2: deletion AAA GAAAAAAAAAAA→ GAAAAAAAA Position: 619187 bp	3: deletion A TAAAAAAAAAA→ TAAAAAAAAA Position: 887064 bp	4 [b]: deletion A GAAAAAAAAA→ GAAAAAAAA Position: 1240663 bp	5 [b]: deletion A GAAAAAAAAAAAA→ GAAAAAAAAAAA Position: 1883778 bp
HMT16	Plasmid pair for HMT18 assembly	/	/	/	/	/
pZR13		NA	NA	NA	NA	NA
HMT18-11	Colonies of assembled	/	Deletion AAA	/	Deletion A	/
HMT18-12	HMT18	/	/	Deletion A	Deletion A	Deletion A
HMT18-13		/	/	/	Deletion A	/
HMT18-14		Deletion T	/	/	/	/

[a] All variants were further subjected to PCR amplification and sequencing.

[b] Variant 4, deletion of one A in GAAAAAAAAAA at position 1240663, and variant 5, deletion of one A in GAAAAAAAAAAAAA at position 1883778 were also observed in Table S6.

Table S8. *De novo* genome sequencing validation of 3-day-passages of HMT18 by long-read Pacbio sequencing and next-generation sequencing.

	Position	Mutation type	HMT18-13 (day 1)	HMT18-13 (day 2) [a]	HMT18-13 (day 3)
1	141168	deletion T	CTTTTTTTTTTTTTT<br>TTTTTTTTTTTTTTT<br>TTTTTTTT	CTTTTTTTTTTTTTT<br>TTTTTTTTTTTTTTT<br>TTTTTTTT	CTTTTTTTTTTTTTT<br>TTTTTTTTTTTTTTT<br>TTTTTTT
2	213762	insertion A	CAAAAAAAAAAAA<br>AAAAAAAAAAAAA<br>AAAA	CAAAAAAAAAAAA<br>AAAAAAAAAAAAA<br>AAAAA	CAAAAAAAAAAAA<br>AAAAAAAAAAAAA<br>AAAAA
3	264011	insertion A	CAAAAAAAAAAAA<br>AAAAAAAAAAAAA<br>A	CAAAAAAAAAAAA<br>AAAAAAAAAAAAA<br>AA	CAAAAAAAAAAAA<br>AAAAAAAAAAAAA<br>AA
4	422068	insertion A	CAAAAAAAAAAAA<br>AAAAAAAAAAAAA<br>AAAAAAA	CAAAAAAAAAAAA<br>AAAAAAAAAAAAA<br>AAAAAAAA	CAAAAAAAAAAAA<br>AAAAAAAAAAAAA<br>AAAAAAAA
5	482655	insertion A	CAAAAAAAAAAAA<br>AAAAAAAAAAAAA<br>AAAA	CAAAAAAAAAAAA<br>AAAAAAAAAAAAA<br>AAAAA	CAAAAAAAAAAAA<br>AAAAAAAAAAAAA<br>AAAAA
6	980498	insertion AT	ATATATATATATATAT<br>ATATATATATATATAT<br>ATATAT	ATATATATATATATAT<br>ATATATATATATATAT<br>ATATATAT	ATATATATATATATAT<br>ATATATATATATATAT<br>ATATATAT
7	1028988	deletion AT	GATATATATATATATA<br>TATATATATATATATA<br>TATATATATATATATA<br>TATATATAT	GATATATATATATATA<br>TATATATATATATATA<br>TATATATATATATATA<br>TATATAT	GATATATATATATATA<br>TATATATATATATATA<br>TATATATATATATATA<br>TATATAT
8	1200004	insertion A	AAAAAAAAAAAAA<br>AAAAAA	AAAAAAAAAAAAA<br>AAAAAA	AAAAAAAAAAAAA<br>AAAAAAA
9	1704607	deletion A	CAAAAAAAAAAAA<br>AAAAAAAAAAAAA<br>AA	CAAAAAAAAAAAA<br>AAAAAAAAAAAAA<br>AA	CAAAAAAAAAAAA<br>AAAAAAAAAAAAA<br>A

10	1860310	deletion A	CAAAAAAAAAAAA AAAAAAAAAAAAA AAAAAAA	CAAAAAAAAAAAA AAAAAAAAAAAAA AAAAAAA	CAAAAAAAAAAAA AAAAAAAAAAAAA AAAAAA

No variants found on the sequences of the day1, day 2 and day 3 samples of HMT18-14.

[a] Variants are marked in red.

Table S9. Strains and plasmids used in this study.

Strains and plasmids	Genotype or description	Source or reference
	Strains	
DH10B	F-, *endA*1, *recA*1, *galE*15, *galK*16, *nupG*, *rpsL*, ΔlacX74, Φ80*lacZ*ΔM15, *araD*139, Δ(*ara*,*leu*)7697, *mcrA*, Δ(*mrr-hsdRMS-mcrBC*), λ-	Host of all original BAC plasmids
MDS42	42 regions deleted from *Escherichia coli* MG1655 genome	Gyorgy Posfai et al.[3]
MT	J23100-*telN* expression cassette inserted in 3845752-38756 bp of *E. coli* MDS42 chromosome	This study
EMT21	Derivative of *E. coli* MDS42, linear chromosome, J23100-*telN*/*tos*, ori_{BAC}, $matP^{\Delta 276\text{-}279}$, kan^r	This study
HMT11	850 kb of human DNA integrated into EMT21 chromosome	This study
HMT12	1.12 Mb of human DNA integrated into EMT21 chromosome	This study
HMT13	1.34 Mb of human DNA integrated into EMT21 chromosome	This study
HMT15	1.55Mb of human DNA integrated into EMT21 chromosome	This study
HMT16	1.96 Mb of human DNA integrated into EMT21 chromosome	This study

HMT18	2.13 Mb of human DNA integrated into EMT21 chromosome	This study
	Plasmids	
pCAS	Overexpression of lambda RED recombination system under induction by arabinose	Yu Jiang et al.[4]
pUZ8002	Helper plasmid of type VI secretion system	Tobias Kieser et al.[5]
pZC1	BAC plasmid CH17-251P17 with *spc-tos-oriT* cassette inserted in 218878- 218879 bp	This study
pZC2	BAC plasmid CH17-133D4 with *tos-oriT-apra* cassette inserted in 215369-14 bp and *hyg* cassette inserted in 61880-61881 bp	This study
pZC3	BAC plasmid CH17-54L2 with *hyg-oriT-tos* cassette inserted in 211783-211800 bp and *kan* cassette inserted in 218552-14 bp	This study
pZC4	BAC plasmid CH17-227G15 with *apra-tos-oriT-spc* cassette inserted in 203865-14 bp	This study
pZC5	BAC plasmid CH17-129O2 with *cm-oriT-tos* cassette inserted in 205289-205290 bp and *spc* cassette inserted in 212058-14 bp	This study
pZC6	BAC plasmid CH17-252L23 with *tos-oriT-apra* cassette inserted in 215771-14 bp	This study
pZC7	BAC plasmid CH17-19I20 with *spc-oriT-tos* cassette inserted in 204158-204181 bp and *apra* cassette inserted in 210933-14 bp	This study
pZC8	BAC plasmid CH17-336K5 with *apra-tos-oriT* cassette inserted in 7578-8459 bp	This study
pZD1	BAC plasmid CH17-293P14 with *oriT-amp-tos* cassette inserted in 1-1500 bp	This study
pZD2	BAC plasmid CH17-136G2 with *kan-tos-oriT* cassette inserted in 7578-8459 bp	This study

pZD3	BAC plasmid CH17-217P20 with *tos-apra-oriT* cassette inserted in 7578-8459 bp	This study
pZD4	BAC plasmid CH17-144K4 with *spc-tos-oriT* cassette inserted in 7578-8459 bp	This study
pZD5	BAC plasmid CH17-35D15 with *oriT-amp-tos* cassette inserted in 1-1500 bp	This study
p1	BAC plasmid CTD-2572O2 with *tos* locus inserted in 193184-193597 bp	This study
p2	BAC plasmid RP11-659B19 with *oriC -spc-oriT-tos* cassette inserted in 169768-178096 bp	This study
p3	BAC plasmid RP11-413L20 with *oriT-apra-tos-*ori_{BAC} cassette inserted in 185198-193526 bp	This study
p4	BAC plasmid RP11-72N10 with *oriT-spc-tos-* ori_{BAC} cassette inserted in 193878-202206 bp	This study
p5	BAC plasmid CTD-3074B5 with *apra-oriT-tos-* ori_{BAC} cassette inserted in 156598-1 bp	This study
p6	BAC plasmid CTD-2195P5 with *spc-oriT-tos-* ori_{BAC} cassette inserted in 146396-153717 bp	This study
p7	BAC plasmid CTD-2366K3 with *apra-oriT-tos-* ori_{BAC} cassette inserted in 145566-152875 bp	This study
p8	Resulting from assembly of three plasmids including p8-1, p8-2 and p8-3	This study
p8-1	BAC plasmid CH17-314I7 with *spc-oriT-tos-*ori_{BAC} cassette inserted in 212350-220380 bp	This study
p8-2	BAC plasmid CTD-3087C18 with *apra-oriT-tos-*ori_{BAC} cassette inserted in 33331-7069 bp	This study
p8-3	Plasmid pUC57-1 with *spc-oriT-tos-* ori_{BAC} cassette inserted in 17170-7171 bp	This study
pUC57-1	106869845-106879844 bp sequence of human chromosome 14 cloned in vector pUC57-Brick	this study

tos: the abbreviation for the *tos* locus which is recognized by TelN nuclease

oriT: the abbreviation for the conjugation transfer initiation sequence

ori_{BAC}: the abbreviation for the replication region of BAC plasmid

oriC: the abbreviation for the replication region of *E. coli* genome

apra: the abbreviation for the apramycin resistant gene

spc: the abbreviation for the spectinomycin resistant gene

kan: the abbreviation for the kanamycin resistant gene

hyg: the abbreviation for the hygromycin resistant gene

cm: the abbreviation for the chloramphenicol resistant gene

amp: the abbreviation for the carbenicillin resistant gene

Materials and Methods.

Strains and plasmids

The strains and plasmids utilized in this study are listed in Table S9. The concentrations of antibiotics used for the *E. coli* strains' cultivation are as following: 34 μg/ml chloramphenicol, 100 μg/ml carbenicillin, 50 μg/ml apramycin, 50 μg/ml kanamycin, 100 μg/ml spectinomycin, 100 μg/ml hygromycin.

The BAC plasmids employed for human DNA assembly were categorized into two groups. The first group comprised 13 BAC plasmids (CH17-251P17, CH17-133D4, CH17-54L2, CH17-227G15, CH17-129O2, CH17-252L23, CH17-19I20, CH17-144K4, CH17-35D15, CH17-136G2, CH17-217P20, CH17-336K5, CH17-293P14), each containing a 194-232 kb sequence of human chromosome 18, collectively covering the region of 3618436-5909203 bp on the short arm of chromosome 18, specifically 18p11.2. The second group of BAC plasmids

contained the sequence of the human IgHV gene cluster, comprising 9 BAC plasmids (CTD-2572O2, RP11-659B19, RP11-413L20, RP11-72N10, CTD-3074B5, CTD-2195P5, CTD-2366K3, CH17-314I7, CTD-3087C18), each encompassing a 150-220 kb sequence of chromosome 14 (excluding CTD-3087C18, which contained only a 26 kb sequence of chromosome 14), collectively covering the region of 105815179-106875815 bp on chromosome 14. To achieve assembly of the complete IgHV, a 10 kb DNA fragment (106869845-106879844 bp) was synthesized by GenScript company and cloned into the vector pUC57-Brick, resulting in the plasmid pUC57-1. All aforementioned BAC plasmids were purchased from the BACPAC Resources Center (BPRC).

Linearization of circular BAC plasmids

The circular BAC plasmids were targeted engineered to be linearized. The targeting cassette comprised five essential components: the left homologous arm (500 bp), the spectinomycin resistance gene, the *tos* sequence, the *oriT* sequence, and the right homology arm (500 bp). Each of these components were separately PCR amplified, wherein a 40 bp overlap between adjacent components was introduced by the primer. Subsequently, these five components were seamlessly assembled into the targeting cassette through fusion PCR (Toyobo, KAO-201X).

Taking the BAC plasmid CH17-251P17 as an example, the process began by electroporating the temperature-sensitive plasmid pCAS into the *E. coli* DH10B strain containing this BAC plasmid (DH10B/CH17-251P17). About 1 μg of targeting cassette DNA was added to 100 μl of electroporation-competent cells of DH10B/CH17-251P17/pCAS. The targeted engineered BAC plasmids were verified by colony PCR. The successfully targeted plasmid CH17-251P17 was designated as pZC1. The plasmid pUZ8002 was then electroporated into the DH10B/pZC1

strain. The plasmid pZC1 was then transferred to the MT strain harboring TelN telomerase by conjugation. TelN nucleases expressed in the MT strain recognized and cleaved the *tos* locus, resulting in the linearization of plasmid pZC1.

Conjugation experiment

For conjugation experiment, 5 OD of overnight culture from the donor strain and 2 OD from the recipient strain were collected. After centrifugation at 5000 rpm for 5 minutes, the cells of both donor and recipient were washed thrice with 1 ml of liquid LB medium. The donor and recipient cells were then resuspended in 50 μl of liquid LB medium each and mixed together. The mixture was plated onto an LB plate and incubated at 37°C for 2 hours. Subsequently, the cells were washed off with 1 ml of liquid LB medium, and 10-fold serial dilutions were prepared. Aliquots of 100 μl from the 10-fold, 100-fold, and 1000-fold dilutions were spread onto LB plates containing antibiotics to select for conjugants. All plates were then incubated at 37°C overnight.

Conjugation facilitated large DNA assembly by the CALBIA method

Taking the assembly of pZC1 and pZC2 as an example, strain MT/pZC2/pUZ8002 acted as the donor for conjugation, while MT/pZC1 served as the recipient. Upon transfer of plasmid pZC2 from the donor to the recipient through conjugation, homologous recombination occurred at the 32 kb overlap region of pZC1 and pZC2, resulting in the formation of a 387 kb linear plasmid named pZR1. Plasmid pZR1 lost the chloramphenicol resistance marker, gained the spectinomycin resistance marker, and retained the apramycin resistance marker.

Preparation of plugs for Pulsed-Field Gel Electrophoresis (PFGE)

To prepare plugs for PFGE, 3-10 single colonies of each conjugation were inoculated into 2 ml

of liquid LB containing corresponding antibiotics and incubated at 37°C overnight. cells equivalent to 1ml of an OD_{600} of 1 were collected from the overnight culture by centrifugation at 12,000 rpm for 1 minute. These cells were then resuspended in 100 µl of TE25S solution (10.3% sucrose, 25 mM Tris• HCl pH 8.0, 25 mM EDTA pH 8.0). Subsequently, 100 µl of 2% low melting point agarose (Biorad, 1613113), melted in TE25S and pre-warmed in a 50°C water bath, was added. After thorough mixing, the mixture was pipetted into the plug mold. Following a 30-minute incubation at 4°C, the plugs were placed in a 2 ml centrifuge tube. 1 ml of NDS solution (1% sodium dodecyl arginine, 0.5 M EDTA pH 8.0, 200 µg/ml proteinase K) was added, and the mixture was incubated at 50°C for 6-16 hours. Subsequently, the plugs underwent four washes with 1 ml of TE10 (10 mM Tris• HCl pH 8.0, 10 mM EDTA pH 8.0), each wash lasting 30 minutes. Finally, the plugs were ready for PFGE.

PFGE

Linear plasmids can be readily detected using PFGE (CHEF-DR III system, Biorad). Various conditions were employed to separate DNA bands of different sizes effectively. For DNA bands ranging from 8 kb to 151 kb, the following parameters were utilized: an initial switch time of 1 second, a final switch time of 25 seconds, voltage set at 6V/cm, angle at 120 degrees, and a total run time of 18 hours. For DNA bands ranging from 200 kb to 1300 kb, the conditions included an initial switch time of 60 seconds, a final switch time of 120 seconds, voltage set at 6V/cm, angle at 120 degrees, and a run time of 24 hours. For larger DNA bands, ranging from 850 kb to 2130 kb, a two-block strategy was implemented. In Block 1, parameters included an initial switch time of 60 seconds, a final switch time of 120 seconds, voltage set at 6V/cm, angle at 120 degrees, and a run time of 12 hours. Block 2 incorporated an initial switch time of 500

seconds, a final switch time of 500 seconds, voltage set at 3V/cm, angle at 106 degrees, and an extended run time of 48 hours.

Construction of the *E. coli* strain EMT21

The replication origin spanning from 3354829 to 3355384 bp on *E. coli* MDS42 chromosome was targeted replaced by a targeting cassette consists of the *tos* site, apramycin resistance gene, and replication origin of BAC. Subsequently, the *tus* gene located at 1365801-1366543 bp on the MDS42 chromosome was deleted, generating the strain EMT5. The integration of the *telN* gene into the genome via PCR-targeting (8248-10204 bp) yielded strain EMT21.

Validation of the genetic stability of Mb-level human DNAs in *E. coli*

A single colony of the test strain was inoculated into 2 ml of liquid LB medium along with the necessary antibiotics. The cell culture was then incubated at 37°C for 24 hours, with continuous shaking. Subsequently, a 2 µl aliquot of the culture was transferred into a fresh 2 ml liquid LB medium supplemented with appropriate antibiotics, and incubated for another 24 hours. This process was repeated on the third day. Cell cultures were sampled every 24 hours. The collected cells (OD_{600}=1) were then embedded in low melt point agarose to form plugs for PFGE.

The assembly of 2.13 Mb human chromosome by iterative integration

Plasmid pZR5 was transferred into strain EMT21 via conjugation and integrated into the linear chromosome using the 5.5 kb of BAC backbone DNA as homologous sequence, resulting in the strain HMT11. Subsequently, plasmids pZR3, pZR12, pZR9, pZR8, and pZR13 were sequentially transferred and iterative integrated using the overlaps sequence carried on each plasmid (ranging from 16-204 kb) as homologous recombination arms. Finally, a total of 2.13 Mb of human chromosome 18 was successfully integrated into the EMT21 chromosome to

generated the strain HMT18.

Multiplex PCR to detect the integrity of assembled human sequence

Multiplex PCR (Toyobo, KFX-201) was used to verify the integrity of the assembled human sequence. Nine pairs of primers were evenly designed to locate on the assembled human DNA. Conjugants exhibiting the detection of all 9 DNA bands were considered to possess complete assembled human DNA sequences. The positive conjugants, as confirmed by multiplex PCR, were then subjected to PFGE analysis.

***In vitro* transcription of guide RNA**

The transcription cassette, designed to produce T7 promoter driven gRNA for BAC backbone cleavage, was cloned into the pSP72 vector to generate the plasmid pSP72-gRNA-BAC. Subsequently, the DNA of the pSP72-gRNA-BAC plasmid was linearized at the *Hin*dIII site. The *in vitro* transcription reaction was set up as follows: NTP buffer mix (10 μl), pSP72-gRNA-BAC (*Hin*dIII) DNA (1 μg), T7 RNA polymerase mix (2 μl), and nuclease-free water was prepared to make a total volume of 30 μl. After thorough mixing, the reaction was briefly centrifuged and then incubated at 37 °C for 18 hours. The HiScribe T7 Quick High Yield RNA Synthesis Kit (NEB, E2050S) was utilized for *in vitro* transcription process. Following transcription, 20 μl of nuclease-free water and 2 μl of DNase I were added to the reaction mixture, and incubated at 37°C for an additional 20 minutes to eliminate template DNA. RNA recovery was carried out using the NucleoSpin RNA Clean-up kit (Cat. No. 740948.50) from Macherey-Nagel.

In plug Cas9 nuclease cleavage

The Cas9 nuclease (NEB, M0386M) was used to release the integrated human DNA from the

E. coli chromosome. About 1/5 of each sample plug was cut and immersed in 200 μl of TE buffer (10 mM Tris•HCl, pH 8.0, 1 mM EDTA, pH 8.0) for 30 minutes, repeating this process once. Next, immerse the plugs in 200 μl of 1x r3.1 buffer (NEB, B6003V) for 30 minutes, again repeating this step. To prepare the Cas9 excision reaction, combine 10 μl of 10x r3.1 buffer, 0.1 μl of Cas9 nuclease (NEB, M0646T), 400 ng of guide RNA, and supplement the reaction volume to 100 μl with nuclease-free water. After thoroughly mixing the Cas9 nuclease reaction, incubated it at room temperature for 10 minutes. Subsequently, added 100 μl of the Cas9 nuclease reaction to each 1/5 plug and incubated at 37°C for 2 hours. Finally, employed the plug for PFGE.

Next-generation sequencing to verify the assembled human DNA sequence

The whole genomes of the ten PFGE-positive HMT18 colonies, six PFGE-positive pG colonies, seven PFGE-positive pZR11 colonies, nine PFGE-positive pZR6 colonies, assembly pairs for each assembly and the strains harboring the original BAC plasmids (CH17-251P17, CH17-133D4, CH17-54L2, CH17-227G15, CH17-129O2, CH17-252L23, CH17-19I20, CH17-144K4, CH17-35D15, CH17-136G2, CH17-217P20, CH17-336K5, CH17-293P14, CTD-2572O2, RP11-659B19, RP11-413L20, RP11-72N10, CTD-2195P5, CTD-2366K3) were sampled for whole genome sequencing (Personal Biotechnology Company, Shanghai, China)). Total DNA from these strains was extracted. The standard Illumina TruSeq Nano DNA LT library preparation protocol (Illumina TruSeq DNA Sample Preparation Guide) was employed to construct the genomic libraries. Subsequently, the samples underwent testing using the Illumina NovaSeq 6000 platform. Alignment of the high-quality data, obtained after filtering, to the reference genome was carried out using the BWA (0.7.17-r1188) mem program, with

alignment parameters set to default settings of BWAMEM. SNPs were detected using GATK software. Furthermore, the Unified Genotyper program within the Genome Analysis TK v3.8 software package was utilized to identify all mutation points within the samples. The stand_call_conf is set to 10, the stand_emit_conf is set to 10, and then Select variants is used to extract the InDel mutation information.

***De novo* sequencing**

Four HMT18 colonies were sampled for *de novo* sequencing. To detect possible variations during continuous passage, two of the colonies were cultured for three days, and samples were collected each day for *de novo* sequencing. All eight samples were sequenced using both the Illumina NovaSeq 6000 and PacBio Revio platforms. The sequencing data obtained from PacBio Revio were assembled using Unicycler and Flye software to obtain contig sequences. The high-quality data from the second-generation sequencing were used to correct the contig using Pilon (Walker BJ et al., 2014), ultimately resulting in the assembly of a complete sequence.

Sequencing data analysis

We used the same analysis strategy applied by Zürcher et al[2]. We assessed the assembly accuracy and structural integrity of the 2.13 Mb assembly in HMT18 using a custom pipeline that integrated 150 bp paired-end Illumina short-read sequencing data and PacBio long-read sequencing data. We corrected the GRCh38/hg38 human reference genome assembly with the input BACs. Then, we aligned the assembly pairs and HMT18 constructs short-read sequencing data against the BAC-corrected reference using the bwa (0.7.12-r1039) mem program. Indels and SNPs in the HMT18 assemblies and the resource assembly pairs were called against the corrected reference. Only SNPs and indels that were exclusively called for the HMT18

assemblies were considered true variants. Variants called in both assembly pairs (HMT16/pZR13) and HMT18 were ruled out, as they were not caused by recombination during assembly. For long or ambiguous variants, we used PCR and sequencing to further validate them. To verify the accuracy of pZR11, pG, and pZR6, we called SNPs and indels in these three megabase plasmids with their corresponding assembly pairs against the corrected reference. Only those variants exclusively found in the assembled pZR11, pG, or pZR6 were regarded as true variants.

Data Availability

All of sequencing data in this paper has been deposited to NCBI with the accession numbers of PRJNA1098915 (HMT18)PRJNA1098934 (EMT21), PRJNA1097768 (CH17-251P17), PRJNA1097783 (CH17-133D4), PRJNA1097800 (CH17-54L2), PRJNA1097810 (CH17-227G15), PRJNA1097835 (CH17-129O2), PRJNA1098439 (CH17-252L23), PRJNA1098493 (CH17-19I20), PRJNA1098503 (CH17-144K4), PRJNA1098576 (CH17-35D15), PRJNA1098870 (CH17-136G2), PRJNA1098873 (CH17-217P20), PRJNA1098885 (CH17-336K5), PRJNA1159494 (pZR3), PRJNA1159547 (pZR5), PRJNA1158873 (nine pZR6 colonies), PRJNA1159598 (pZR7), PRJNA1160229 (pZR10), PRJNA1161133 (seven pZR11 colonies), PRJNA1159480 (HMT16), PRJNA1159483 (pZR13), PRJNA1158507 (eighteen HMT18 samples), PRJNA1162302 (CTD-2572O2, RP11-659B19, RP11-413L20, RP11-72N10, CTD-3074B5, CTD-2195P5, CTD-2366K3), PRJNA1160705 (p8), PRJNA1160241 (pF), PRJNA1161033 (six pG colonies).

References

1 He, B. *et al.* YLC-assembly: large DNA assembly via yeast life cycle. *Nucleic Acids Research*, doi:10.1093/nar/gkad599 (2023).

2 Zürcher, J. F. *et al.* Continuous synthesis of *E. coli* genome sections and Mb-scale human DNA assembly. *Nature* **619**, 555-+, doi:10.1038/s41586-023-06268-1 (2023).

3 Pósfai, G. *et al.* Emergent properties of reduced-genome. *Science* **312**, 1044-1046, doi:10.1126/science.1126439 (2006).

4 Jiang, Y. *et al.* Multigene Editing in the Genome via the CRISPR-Cas9 System. *Appl Environ Microb* **81**, 2506-2514, doi:10.1128/Aem.04023-14 (2015).

5 Kieser, T. *et al. Practical Streptomyces Genetics: A Laboratory Manual*. (2000).
